# The prevalence of obesity and influence of early life and behavioral factors on obesity in Chinese children in Guangzhou

**DOI:** 10.1186/s12889-016-3599-3

**Published:** 2016-09-09

**Authors:** Ting Zhang, Li Cai, Lu Ma, Jin Jing, Yajun Chen, Jun Ma

**Affiliations:** 1 Department of Maternal and Child Health, School of Public Health, Sun Yat-Sen University, Guangzhou, 510080 Guangdong China; 2 Institute of Child and Adolescent Health, School of Public Health, Peking University, Beijing, 100191 China

**Keywords:** Childhood obesity, Early life factor, Dietary intake, Sedentary time, Weight-loss practice

## Abstract

**Background:**

Childhood obesity has become a public health concern in many countries. In Southern China, the prevalence of childhood obesity increased from 6.2 to 7.5 % between 2007 and 2011. This study aimed to report the current prevalence of overweight and obesity, analyzed the early life and behavioral determinants of obesity, and investigated the weight-loss practices among Chinese children in Guangzhou.

**Methods:**

Three thousand seven hundred sixty-six primary school students aged 7–12 years were recruited in Guangzhou, China in 2013. Questionnaires were used to assess (1) early life factors: birth weight, delivery mode, gestational age and feeding patterns; (2) behavioral factors: dietary intake, eating speed, sedentary time, physical activities and sleep duration; and (3) weight-loss practices: improving diet, increasing exercise, taking weight-loss drugs and undergoing a diet. The criteria of Working Group of Obesity in China were applied to classify overweight and obesity based on measured weight and height. Multivariable logistic regression analyses were performed to examine the determinants of overweight/obesity and adoption of weight-loss practices.

**Results:**

The prevalence of childhood overweight and obesity were 11.2 and 10.0 %, respectively. High birth weight (≥4.0 kg versus 2.5 ~ 4.0 kg, odd ratio [OR]: 2.34; 95 % confidence interval [CI]: 1.53–3.58), sugar-sweetened beverages (SSBs) intake (OR: 1.39; 95 % CI: 1.05–1.85), vegetable intake (OR: 1.12; 95 % CI: 1.01–1.24), and doing homework (OR: 1.24; 95 % CI: 1.08–1.43) were positively associated with obesity. Eating speed faster than peers was positively associated with obesity and yielded the highest OR (versus “as fast as peers”, OR: 3.18; 95 % CI: 2.28–4.44). Approximately 57, 81 and 87 % of normal-weight, overweight and obese children, respectively, reported weight-loss practices. Self-perception of weight status presented as the strongest determinant for weight-loss practices.

**Conclusions:**

The prevalence of overweight and obesity were high in Chinese children in Guangzhou, and both were higher than previous level in 2011. Further research should address the unhealthy dietary (e.g. SSBs intake, fast eating speed) and sedentary behaviors (e.g. doing homework) of these children. Moreover, an accurate perception of body weight can help promote the adoption of weight-loss practices in overweight and obese children.

## Background

Childhood obesity has become a public health concern in many countries [1]. Worldwide, the prevalence of overweight and obesity combined rose by 47.1 % for children between 1980 and 2013 [1]. In 2013, approximately 23.8 and 22.6 % of boys and girls in developed countries were overweight or obese, while 12.9 and 13.4 % were observed in developing countries [1]. With rapid economic development over the past decades, China has also witnessed an increase in childhood obesity [2, 3]. In 2010, it was estimated that 9.9 and 5.1 % of school-age children and adolescents in China were overweight and obese, respectively, which amounted to approximately 30.4 million individuals [4]. Moreover, the prevalence of obesity in some areas of China has been catching up with that in developed countries. For example, the prevalence of obesity in Shandong Province in 2010 reached 15.8 and 7.1 % in boys and girls, respectively [5].

Childhood obesity is associated with an increased risk of cardiovascular disease and type 2 diabetes mellitus [6] and may persist into adulthood [7]. Given its high prevalence and risk, childhood obesity requires immediate and effective interventions. Health officials must continue to monitor and provide insights into the underlying modifiable determinants of childhood obesity. Previous studies suggested that the determinants of obesity in children varied across different regions, ethnicities and populations [8–16]. In China, several studies reported that behavioral factors such as dietary intake [8, 11, 15], fast eating speed [15], sedentary behaviors [8, 11, 13, 16], physical inactivity [8], and short sleep time [11, 13] were closely associated with obesity risk in children. In recent years, an increasing number of studies have focused on the fetal and early life origins of subsequent obesity (e.g., birth weight, delivery mode and breastfeeding pattern). For instance, a meta-analysis revealed that high birth weight (>4.0 kg) was associated with increased risk of obesity from childhood to early adulthood [17]. A Chinese birth cohort study demonstrated that caesarean delivery modestly increased overweight risk at 4 to 7 years of age compared with vaginal delivery [18]. A longitudinal study suggested that breastfeeding was associated with decreased risk of overweight and obesity among schoolchildren in Japan [19]. In China, the early life factors associated with obesity were mainly studied among younger-aged children [18, 20, 21], but were less understood in primary school children.

Guangzhou is one of the most developed cities in southern China, where the prevalence of obesity in children (aged 7–18 years) increased from 6.0 % in 2007 to 6.6 % in 2011 [22]. Another study also reported that the prevalence of obesity among 7–12-year-old children in Guangzhou increased from 6.2 to 7.5 % between 2007 and 2011 [23]. Further studies are needed to monitor this trend. Previous studies demonstrated that the prevalence of overweight and obesity among children in Guangzhou were lower than other large coastal cities in China [4, 24]. The climate and lifestyle habits in southern China are markedly different from that in other regions. For example, due to the warm and humid climate and cultural difference, most people in this area tend to consume less greasy or strongly flavored food. So the risk factors of childhood obesity may be quite different from those of other areas. However, limited data on the determinants of childhood overweight or obesity has been obtained from this area. Research focusing on the determinants of childhood obesity in this area can provide guidance for effective obesity prevention and intervention programs. Another question is how children would be engaged in weight-loss practices and what factors would be related to their adoption. So far, the picture of weight-loss practices (e.g., exercising to lose weight and going on a diet) among Chinese primary school children is unknown. Our study adds to previous literature by investigating children’s engagement in weight-loss practices, which may contribute to prevention of unhealthy weight-loss practices in primary school children. Therefore, the present study aims to assess the current extent of the obesity epidemic, investigate the early life and behavioral factors influencing such phenomenon, and analyze the weight-loss practices of primary school children in Guangzhou, China.

## Methods

### Study design and data source

This cross-sectional study is the baseline survey of a school-based obesity intervention project that targeted Chinese children and adolescents (“Development and Application of Student Critical Diseases Prevention and Control Technology and Related Standards”) (Trial registration: January 22, 2015; Registration number: NCT02343588) [25, 26]. The project was approved by the Ethical Committee of Peking University. All participating students and their parents voluntarily signed informed consent forms.

### Sampling method and participant recruitment

With multistage random cluster sampling method, the participants were recruited in urban areas of Guangzhou. Firstly, we selected four urban districts from ten districts of Guangzhou using judgment sampling method. Secondly, one or two primary schools were randomly selected from each of the four districts. Probability proportional to size sampling method was used in this stage and a total of seven schools were selected. The principals were sent invitation letters, information sheets and presentations that outlined the research details. After the principals’ permission, all students from the second to the fifth grade were invited to participate, excluding those aged below 7 years old or were diagnosed with visceral diseases, abnormal growth and development, physical abnormality or obesity induced by endocrine diseases or drugs. Consent forms for the physical examination and questionnaire survey were given to each student. The children were advised to discuss the research with their parents and then return the consent forms to the school if they and their parents were willing to participate. A total of 4942 students agreed to participate in the study. The response rates were 100 % (7/7) for schools and 78.4 % (4942/6300) for students. Participants who did not take questionnaires (*n* = 690) or physical examination (*n* = 473), or had missing information about their age (*n* = 1), gender (*n* = 0), or body mass index (BMI) (*n* = 12) were excluded from the study, leaving 3766 participants in the sample. The study was conducted between September and November 2013.

### Questionnaire survey

To ensure that each question was explained similarly to all the participants, the student and parent questionnaires were administered to the whole class during school hours by the same investigators, who were trained postgraduates. A teacher supervised and assisted the survey procedure in each class. Students in grades 4–5 filled out the student questionnaire by themselves in class, whereas students in grades 2–3 answered the student questionnaire under parents’ guidance at home. The parent questionnaire and explanatory cover letters were delivered by children to their parents and returned to the researchers after 3 days. All questionnaires were checked for integrity upon return. The questionnaires with missing values were resubmitted to children or their parents to be filled out again. Children’s birth date in the questionnaire was checked for logicality, which should be between January 2001 and November 2006.

#### Socio-demographic characteristics and other measures

Socio-demographic information included children’s birth date, gender and only child or not in the student questionnaire, and parents’ educational level, occupation and monthly household income in the parent questionnaire. Parents reported their own current weight (kg) and height (cm) to evaluate weight status. Weight-loss practices were assessed by asking children the four questions: “Have you tried to lose weight in the following ways over the past 3 months? (1) Improving diet (i.e., eating more fruit or vegetable but less high-energy snacks), (2) Increasing exercise, (3) Taking weight-loss drugs, (4) Undergoing a diet (i.e., not eating staple foods like rice or noodles)”. The responses for each behavior were “yes” or “no”. Children’s self-perception of weight status was assessed by asking: “How do you feel about your current weight status?” with the five response options: “very thin”, “rather thin”, “average”, “rather fat” and “very fat”. The responses were categorized into: perceived underweight (“very thin” and “rather thin”), normal weight (“average”) and overweight (“very fat” and “rather fat”).

#### Early life factors

Parents were asked about the participating child’s birth weight (kg), mode of delivery (vaginal delivery or cesarean delivery), gestational age (week), feeding patterns (breastfeeding or not), and breastfeeding duration (month) in the parent questionnaire.

#### Behavioral factors

Behavioral factors included 8 items for dietary intake, 1 item for eating speed, 3 items for sedentary time, 3 items for physical activities, and 1 item for sleep duration. Dietary intake included the consumption of fruits, vegetables, meat products, breakfast, sugar-sweetened beverages (SSBs), high-energy snacks (e.g., chocolates and candies), fried food (e.g., fried chicken and fried potatoes), and western fast food (e.g., KFC and McDonald’s). Children reported the frequency (days) and amount (servings) of fruits, vegetables, meat/meat products and SSBs intake over the past 7 days. The average daily intake of single food was estimated as follows: average daily intake = [days × (amount in each of those days)]/7. Eating speed was evaluated by asking: “What do you think about your eating speed compared with peers?” The three response options were “slower than peers”, “as fast as peers” and “faster than peers”.

Sedentary behavior included doing homework, viewing television and using a computer (including playing video games). Children were asked about the average amount of time (hours and minutes) they spent daily on the three behaviors over the past 7 days. Physical activities included vigorous-intensity physical activities (aerobic activities that significantly increase heart rate and breathing, e.g., running, basketball, football and swimming, etc.), moderate-intensity physical activities (aerobic activities that increase heart rate and breathing to some extent, e.g., cycling, table tennis, badminton and calisthenics, etc.), and walking. Children reported the frequency (days) and duration (hours and minutes in each of those days) of these activities over the past 7 days. The average daily time of physical activities were calculated as follows: average daily time = [days × (amount in each of those days)]/7. Sleep duration was assess by asking: “How long do you sleep every day?” with four response options: “<7.0 h”, “7.0 ~ 8.9 h”, “9.0 ~ 11.0 h”, and “>11.0 h”.

### Physical examination

Physical examination was conducted 3 days after questionnaire survey. The children underwent a thorough medical examination to ensure that they were free from visceral diseases and abnormal obesity induced by endocrine diseases or drugs. Height (cm) and weight (kg) were measured by a team of trained technicians following a standardized procedure. Height was measured using the portable stadiometer (model TZG, China) (with 0.1 cm precision), with the subjects standing straight and barefoot. Weight was measured using the lever type weight scale (model RGT-140, China) (weighing 120 kg with 0.1 kg precision). The subjects were asked to use the restroom beforehand and stand on the scale wearing only their underwear. Both height and weight were measured twice and averaged. About 5 % of the children would be rechecked for height and weight. If the error exceeds 10 %, all of the students have to be measured again.

BMI was calculated by dividing weight in kilograms by height in meters squared (kg/m^2^). Children’s overweight and obesity were defined by using the Working Group of Obesity in China (WGOC) [27] criteria, the cut-off points of which are 85th and 95th percentiles of BMI, respectively (Overweight: 85th percentile ≤ BMI < 95th percentile; Obesity: 95th percentile ≤ BMI). Children’s underweight was defined based on the age-gender-specific BMI cutoffs of malnutrition for Chinese children [28]. Parents’ BMI was calculated using their self-reported height and weight. Parental overweight was defined as BMI ≥ 24 kg/m^2^ to <28 kg/m^2^, whereas obesity was defined as BMI ≥ 28 kg/m^2^ in accordance with the criteria for Chinese adults [29].

### Statistical analysis

Calculations were conducted using IBM SPSS software version 19.0. Descriptive statistics were used to characterize the study population. Continuous variables were presented as mean ± standard deviation (SD), whereas categorical variables were presented as percentages. For weight status, the figures for “overweight” excluded those for “obese” and the figures for “normal weight” excluded those for “underweight”. Differences of weight status between two items or more were evaluated by Mann-Whitney U tests or Kruskal-Wallis tests, respectively. Multinomial logistic regression analyses were performed to assess the associations of early life and behavioral factors with overweight/obesity. Such association was initially assessed in the crude model. The second set of model (adjusted model) was adjusted for age, gender and other socio-demographic factors (i.e., only child or not, paternal and maternal educational level, paternal and maternal occupation and monthly household income). Differences in the adoption of weight-loss practices among normal-weight, overweight and obese children were evaluated by Pearson Chi-square tests. Binary logistic regression analyses were performed to assess associated factors of the adoption of weight-loss practices among socio-demographic characteristics and children’s perceptions of weight status. Independent variable screening was performed using the enter method. A two-sided *P* < 0.05 indicated statistical significance.

## Results

Of the 3766 children, the mean age was 8.5 (SD 1.2) years old. Almost half of the children were boys (50.5 %) and most were only child (75.3 %). 1567 (42.6 %) fathers and 1352 (36.9 %) mothers had an education level of college or above. And 1362 (37.7 %) fathers and 1337 (37.0 %) mothers were working in commerce and service industry. Nearly a quarter (23.2 %) of children’s family had a monthly household income of below 8000 RMB, whereas 36.7 % refused to disclose their income (Table 1).

**Table 1:** Characteristics of the population and distribution of weight status in children

Characteristics	Proportion *n* (%)	Distribution of weight status (%)				*P* ^a^
		Underweight	Normal weight	Overweight	Obese	
*Total*	3766 (100 %)	14.5 %	64.4 %	11.2 %	10.0 %	
*Socio-demographics*						
Age (year)						**0.002**
7	962 (25.5)	15.4	67.4	9.0	8.2	
8	998 (26.5)	16.1	62.2	12.5	9.1	
9	869 (23.1)	13.5	63.3	12.0	11.3	
10	840 (22.3)	12.1	64.6	11.4	11.8	
11 ~ 12	97 (2.6)	19.6	63.9	8.2	8.2	
Gender						**<0.001**
Boys	1900 (50.5)	12.1	59.7	14.3	13.8	
Girls	1866 (49.5)	17.0	69.1	7.9	6.0	
Only child or not						0.993
Yes	2792 (75.3)	14.7	64.1	11.2	10.0	
No	918 (24.7)	14.1	65.3	11.3	9.4	
Paternal educational level						0.394
Junior high school or below	371 (10.1)	11.3	67.4	11.1	10.2	
Senior high school	900 (24.5)	15.1	63.4	11.4	10.0	
Junior college	837 (22.8)	16.7	62.4	11.8	9.1	
College or above	1567 (42.6)	13.8	65.0	11.0	10.3	
Maternal educational level						0.486
Junior high school or below	461 (12.6)	15.2	62.5	13.0	9.3	
Senior high school	922 (25.2)	13.0	65.0	11.1	11.0	
Junior college	930 (25.4)	16.6	61.9	12.4	9.1	
College or above	1352 (36.9)	13.9	65.7	10.3	10.1	
Paternal occupation						0.626
Commerce and services	1362 (37.7)	13.4	65.0	11.8	9.8	
Professional and technical	752 (20.8)	14.8	65.8	9.4	10.0	
Administrator and clerk	741 (20.5)	15.8	62.9	10.9	10.4	
Other	757 (21.0)	14.4	63.5	12.5	9.5	
Maternal occupation						0.111
Commerce and services	1337 (37.0)	13.5	64.2	11.8	10.5	
Professional and technical	844 (23.4)	15.5	65.4	10.5	8.5	
Administrator and clerk	444 (12.3)	12.6	64.9	11.3	11.3	
Housewife	434 (12.0)	12.9	65.4	11.3	10.4	
Other	555 (15.4)	17.5	61.4	11.4	9.7	
Monthly household income						0.394
< 8000 RMB	844 (23.2)	14.8	64.3	10.7	10.2	
8000 to 15,000 RMB	770 (21.2)	12.9	65.1	12.7	9.4	
≥ 15,000 RMB	689 (18.9)	14.8	66.6	10.4	8.1	
Refused to answer	1334 (36.7)	15.2	63.3	10.9	10.6	
*Early life factors of children*						
Birth weight (kg)						**<0.001**
< 2.5	171 (4.8)	22.8	63.2	7.6	6.4	
2.5 ~ 4.0	3166 (89.0)	14.6	64.6	11.2	9.5	
≥ 4.0	222 (6.2)	7.2	56.3	16.2	20.3	
Mode of delivery						**0.012**
Vaginal delivery	1692 (46.4)	15.1	65.9	10.2	8.8	
Cesarean delivery	1956 (53.6)	14.2	62.9	12.1	10.9	
Gestational age (week)						0.181
< 37	165 (4.6)	15.8	57.6	13.9	12.7	
37 ~ 42	3378 (93.9)	14.4	64.7	11.0	9.8	
≥ 42	56 (1.6)	7.1	66.1	16.1	10.7	
Feeding patterns						0.863
Breastfeeding	2965 (80.0)	14.4	64.5	11.2	9.9	
Not breastfeeding	739 (20.0)	14.7	64.1	11.5	9.6	
Breastfeeding duration (month)						0.197
< 4.0	1277 (38.3)	15.6	63.5	11.6	9.3	
4.0 ~ 5.9	447 (13.4)	13.2	70.2	9.6	6.9	
6.0 ~ 7.9	691 (20.7)	14.9	63.5	11.7	9.8	
≥ 8.0	920 (27.6)	13.4	63.9	11.1	11.6	
Paternal weight status						**<0.001**
Normal weight	1986 (55.7)	19.3	64.7	9.1	6.8	
Overweight	1260 (35.3)	9.6	65.3	13.0	12.1	
Obese	319 (8.9)	4.1	57.4	19.4	19.1	
Maternal weight status						**<0.001**
Normal weight	3084 (85.4)	15.3	65.7	10.7	8.3	
Overweight	447 (12.4)	9.6	57.7	14.8	17.9	
Obese	80 (2.2)	7.5	50.0	16.3	26.3	

Bold text: *P* value was significant at 0.05 level

^a^Differences of weight status between two or more items of characteristics were evaluated by performing Mann–Whitney U tests or Kruskal-Wallis tests, respectively

On the basis of the WGOC criteria [27], the prevalence of overweight and obesity among the participating children were 11.2 % (14.3 % in boys and 7.9 % in girls) and 10.0 % (13.8 % in boys and 6.0 % in girls), respectively. A higher prevalence of overweight and obesity was observed among older children or boys (*P* < 0.01). An increased prevalence of overweight and obesity was also observed among children with high birth weight (≥4.0 kg) or delivered by caesarean section (*P* < 0.05). Regarding the breastfeeding duration, the lowest prevalence of overweight and obesity was found among children who were breastfed for 4.0 ~ 5.9 months, though there was no significant difference of weight status between different breastfeeding duration (*P* = 0.20). Furthermore, a higher prevalence of overweight and obesity was observed among children who had an overweight or obese father or mother (*P* < 0.001) (Table 1). Overweight or obese children consumed more meat products, SSBs and fried food per day over the past week and were more likely to have faster eating speed than peers compared with underweight or normal-weight children (*P* < 0.05). Overweight or obese children also had longer daily time of doing homework and using a computer than their counterparts (*P* < 0.01). By contrast, no statistical difference was observed among physical activities and sleep duration between different weight status categories (Table 2).

**Table 2:** Behavioral factors of children by weight status

Behavioral factors	Underweight	Normal weight	Overweight	Obese	*P* ^d^
Dietary behavior, mean (SD)					
Fruits (servings ^a^/d)	1.24 (1.07)	1.36 (1.15)	1.37 (1.25)	1.39 (1.14)	0.142
Vegetables (servings ^a^/d)	1.86 (1.60)	1.90 (1.58)	2.02 (1.71)	2.00 (1.71)	0.325
Meat products (serving ^b^/d)	1.46 (1.34)	1.47 (1.34)	1.61 (1.63)	1.65 (1.65)	**0.037**
SSBs (cup ^c^/d)	0.19 (0.36)	0.21 (0.42)	0.31 (0.58)	0.30 (0.66)	**<0.001**
Breakfast (d/week)	6.84 (0.79)	6.83 (0.84)	6.78 (0.99)	6.79 (0.97)	0.568
High-energy snacks (d/week)	1.64 (1.75)	1.55 (1.70)	1.54 (1.68)	1.40 (1.61)	0.222
Fried food (time/week)	0.56 (1.07)	0.59 (1.07)	0.75 (1.21)	0.67 (1.12)	**0.019**
Western fast food (time/week)	1.24 (2.30)	1.42 (2.61)	1.55 (2.34)	1.53 (2.94)	0.225
Eating speed, n (%)					**<0.001**
Slower than peers	211 (39.0)	666 (27.7)	60 (14.4)	45 (12.2)	
As fast as peers	269 (49.7)	1285 (53.5)	201 (48.3)	145 (39.3)	
Faster than peers	61 (11.3)	452 (18.8)	155 (37.3)	179 (48.5)	
Sedentary behavior, mean (SD)					
Doing homework (hour/d)	1.98 (1.15)	1.90 (1.04)	2.04 (1.21)	2.07 (1.20)	**0.007**
Viewing television (hour/d)	0.73 (0.85)	0.79 (0.95)	0.84 (0.91)	0.89 (0.96)	0.057
Using a computer (hour/d)	0.45 (0.69)	0.47 (0.75)	0.60 (0.88)	0.60 (0.81)	**<0.001**
Physical activities, mean (SD)					
Vigorous-intensity physical activity (hour/d)	0.51 (0.63)	0.56 (0.60)	0.60 (0.65)	0.60 (0.76)	0.177
Moderate-intensity physical activity (hour/d)	0.41 (0.50)	0.46 (0.60)	0.48 (0.80)	0.48 (0.67)	0.234
Walking (hour/d)	0.78 (0.97)	0.86 (1.26)	0.86 (1.26)	0.83 (1.03)	0.545
Sleep duration (hour/d), n (%)					0.593
< 7.0	30 (5.9)	125 (5.5)	21 (5.5)	16 (4.6)	
7.0 ~ 8.9	309 (60.4)	1313 (57.7)	234 (60.8)	215 (62.1)	
9.0 ~ 11.0	167 (32.6)	798 (35.1)	120 (31.2)	110 (31.8)	
> 11.0	6 (1.2)	39 (1.7)	10 (2.6)	5 (1.4)	

Bold text: *P* value was significant at 0.05 level

^a^A serving of fruit or vegetable is equivalent to 100 g

^b^A serving of meat products is equivalent to 75 g

^c^A cup is equivalent to 250 ml

^d^Differences of continuous and categorized variables between different weight status groups were evaluated by performing ANOVAs and Pearson chi-square tests, respectively

The associations between early life and behavioral factors and overweight/obesity were examined. Among the early life factors, high birth weight (≥4.0 kg versus 2.5 ~ 4.0 kg) was positively associated with obesity (adjusted odd ratio [OR]: 2.34; 95 % confidence interval [CI]: 1.53–3.58). Gestational age < 37 weeks (versus 37 ~ 42 weeks) was positively associated with overweight (adjusted OR: 1.75; 95 % CI: 1.01–3.05) (Table 3). In terms of behavioral factors, SSBs intake was positively associated with overweight (adjusted OR: 1.37; 95 % CI: 1.03–1.83) whereas sleep duration as 9.0 ~ 11.0 h was negatively related to overweight (versus 7.0 ~ 8.9 h, adjusted OR: 0.60; 95 % CI: 0.43–0.84). Meanwhile, vegetable intake, SSBs intake (adjusted OR: 1.39; 95 % CI: 1.05–1.85) and homework time (adjusted OR: 1.24; 95 % CI: 1.08–1.43) were positively associated with obesity. Eating speed faster than peers (versus “as fast as peers”) was significantly positively associated with both overweight and obesity and yielded the highest ORs (adjusted OR: 1.76; 95 % CI: 1.27–2.43 and adjusted OR: 3.18; 95 % CI: 2.28–4.44, respectively). By contrast, eating speed slower than peers (versus “as fast as peers”) showed negative association with overweight and obesity (adjusted OR: 0.55; 95 % CI: 0.36–0.84 and adjusted OR: 0.55; 95 % CI: 0.34–0.91, respectively). Physical activities showed no relation with overweight or obesity (Table 4).

**Table 3:** Associations of early life factors with overweight/obesity

Early life factors	Overweight		Obese	
	Crude model	Adjusted model^a^	Crude model	Adjusted model^a^
Birth weight (reference: 2.5 ~ 4.0 kg)				
< 2.5 kg	0.51 (0.26, 1.02)	0.49 (0.23, 1.01)	0.61 (0.30, 1.25)	0.68 (0.32, 1.43)
≥ 4.0 kg	1.49 (0.95, 2.33)	1.42 (0.89, 2.26)	**2.64 (1.78, 3.92)** ^**^	**2.34 (1.53, 3.58)** ^**^
Mode of delivery (reference: vaginal delivery)				
Cesarean delivery	1.19 (0.94, 1.50)	1.17 (0.92, 1.50)	1.17 (0.91, 1.50)	1.18 (0.90, 1.55)
Gestational age (reference: 37 ~ 42 weeks)				
< 37 weeks	**1.96 (1.15, 3.33)** ^*^	**1.75 (1.01, 3.05)** ^*^	1.71 (0.95, 3.08)	1.34 (0.71, 2.54)
≥ 42 weeks	1.67 (0.75, 3.72)	1.39 (0.58, 3.30)	0.81 (0.28, 2.38)	0.59 (0.17, 2.04)
Feeding patterns (reference: not breastfeeding)				
Breastfeeding	1.08 (0.75, 1.56)	1.06 (0.72, 1.57)	0.98 (0.66, 1.47)	1.12 (0.73, 1.72)
Breastfeeding duration (reference: < 4.0 months)				
4.0 ~ 5.9 months	0.66 (0.43, 1.03)	0.68 (0.43, 1.08)	**0.61 (0.37, 1.00)** ^*^	0.62 (0.37, 1.03)
6.0 ~ 7.9 months	0.94 (0.65, 1.37)	1.01 (0.68, 1.50)	0.93 (0.62, 1.40)	0.93 (0.60, 1.43)
≥ 8.0 months	0.88 (0.61, 1.25)	0.87 (0.60, 1.27)	1.14 (0.78, 1.65)	1.04 (0.70, 1.55)

Bold text: OR was significant at 0.05 level

Values were represented as OR and 95 % CIs derived from multinomial logistic regression analyses. The reference level for dependent variable was “normal weight”, which excluded the underweight children

^a^Adjusted for age, gender, only child or not, paternal and maternal educational level, paternal and maternal occupation and monthly household income

^*^ *P* < 0.05; ^**^ *P* < 0.01

**Table 4:** Associations of behavioral factors with overweight/obesity

Behavioral factors	Overweight		Obese	
	Crude model	Adjusted model ^a^	Crude model	Adjusted model ^a^
Dietary intake				
Fruits (serving/d)	0.98 (0.86, 1.11)	0.99 (0.87, 1.13)	0.94 (0.82, 1.08)	0.96 (0.83, 1.11)
Vegetables (serving/d)	1.05 (0.95, 1.15)	1.05 (0.95, 1.16)	1.08 (0.98, 1.19)	**1.12 (1.01, 1.24)** ^*^
Meat products (serving/d)	1.01 (0.91, 1.12)	0.97 (0.87, 1.08)	1.00 (0.90, 1.12)	0.96 (0.85, 1.08)
SSBs (cup/d)	**1.32 (1.00, 1.74)** ^*^	**1.37 (1.03, 1.83)** ^*^	**1.42 (1.09, 1.86)** ^*^	**1.39 (1.05, 1.85)** ^*^
Breakfast (d/week)	0.97 (0.83, 1.13)	0.93 (0.79, 1.09)	0.98 (0.83, 1.16)	1.01 (0.83, 1.23)
High-energy snacks (d/week)	0.94 (0.85, 1.03)	0.91 (0.83, 1.01)	0.95 (0.87, 1.05)	0.97 (0.87, 1.07)
Fried food (time/week)	1.11 (0.97, 1.26)	1.10 (0.96, 1.26)	1.05 (0.91, 1.20)	1.07 (0.93, 1.24)
Western fast food (time/week)	1.00 (0.95, 1.06)	1.01 (0.95, 1.06)	0.99 (0.94, 1.05)	0.97 (0.91, 1.04)
Eating speed (reference: as fast as peers)				
Slower than peers	**0.60 (0.40, 0.89)** ^*^	**0.55 (0.36, 0.84)** ^**^	**0.58 (0. 37, 0.92)** ^*^	**0.55 (0.34, 0.91)** ^*^
Faster than peers	**2.01 (1.48, 2.73)** ^**^	**1.76 (1.27, 2.43)** ^**^	**3.59 (2.64, 4.89)** ^**^	**3.18 (2.28, 4.44)** ^**^
Sedentary time				
Doing homework (hour/d)	1.03 (0.91, 1.17)	1.04 (0.90, 1.19)	**1.18 (1.04, 1.33)** ^*^	**1.24 (1.08, 1.43)** ^**^
Viewing television (hour/d)	1.00 (0.83, 1.19)	1.02 (0.84, 1.23)	1.05 (0.88, 1.24)	1.10 (0.91, 1.32)
Using a computer (hour/d)	1.03 (0.84, 1.26)	1.01 (0.81, 1.26)	1.12 (0.92, 1.36)	1.04 (0.84, 1.30)
Physical activity				
Vigorous physical activity (hour/d)	0.99 (0.77, 1.27)	0.97 (0.75, 1.26)	1.06 (0.83, 1.36)	0.96 (0.73, 1.25)
Moderate physical activity (hour/d)	1.00 (0.79, 1.28)	1.03 (0.80, 1.33)	0.97 (0.75, 1.26)	1.00 (0.76, 1.31)
Walking (hour/d)	0.94 (0.84, 1.06)	0.94 (0.83, 1.07)	0.90 (0.79, 1.03)	0.92 (0.80, 1.06)
Sleep duration (reference: 7.0 ~ 8.9 h)				
< 7.0 h	0.83 (0.45, 1.53)	0.84 (0.44,1.57)	0.84 (0.44,1.60)	0.93 (0.47, 1.85)
9.0 ~ 11.0 h	**0.62 (0.46, 0.85)** ^**^	**0.60 (0.43,0.84)** ^**^	0.82 (0.60,1.12)	0.87 (0.62, 1.22)
> 11.0 h	1.57 (0.66, 3.74)	1.69 (0.69,4.16)	0.73 (0.23,2.32)	0.88 (0.26, 2.91)

Bold text: OR was significant at 0.05 level

Values were represented as OR and 95 % CIs derived from multinomial logistic regression analyses. The reference level for dependent variable was “normal weight”, which excluded the underweight children

^a^Adjusted for age, gender, only child or not, paternal and maternal educational level, paternal and maternal occupation and monthly household income

^*^ *P* < 0.05; ^**^ *P* < 0.01 ﻿

**Fig. 1:**
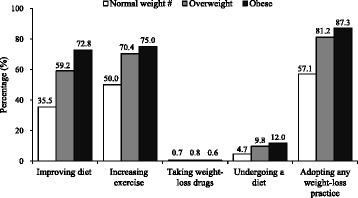
Percentages of adopting weight-loss practices among normal-weight, overweight and obese children. ^#^ The normal-weight group did not include the underweight children

Regarding the adoption of weight-loss practices, there were 40.9, 53.5, 0.7 and 5.9 % children who tried to lose weight through improving diet, increasing exercise, taking weight-loss drugs and undergoing a diet, respectively. Overweight and obese children demonstrated a higher tendency to improve their diet, perform more exercises or undergo a diet than normal-weight children (*P* < 0.001) (Fig. 1). Among the socio-demographic characteristics and personal weight perception, age was positively associated with adopting any of the four weight-loss practices in all three BMI categories. Normal-weight girls were more likely to adopt weight-loss practice than their counterparts. Personal perceived overweight and obese were strongly positively associated with adopting weight-loss practice in obese children (OR: 4.34; 95 % CI: 1.45–12.98 and OR: 5.55; 95 % CI: 1.35–22.92, respectively) and the total population (Table 5).

**Table 5:** Associated factors of adopting any of the four weight-loss practices stratified by actual weight status

Characteristics	Adopting weight-loss practice			Total
	Normal weight ^b^	Overweight	Obese	
Age	**1.41 (1.30, 1.53)** ^**^	**1.77 (1.31, 2.39)** ^**^	**2.59 (1.68, 4.00)** ^**^	**1.40 (1.31, 1.50)** ^**^
Gender (reference: boy)				
Girl	**1.29 (1.07, 1.55)** ^**^	1.93 (0.99, 3.74)	1.60 (0.64, 3.99)	**1.19 (1.02, 1.38)** ^*^
Only child (reference: no)				
Yes	0.87 (0.69, 1.10)	1.09 (0.53, 2.24)	0.87 (0.29, 2.58)	0.87 (0.72, 1.06)
Paternal educational level (reference: junior high school or below)				
Senior high school	1.21 (0.83, 1.76)	1.35 (0.40, 4.52)	1.43 (0.26, 7.69)	1.23 (0.89, 1.70)
Junior college	0.94 (0.62, 1.40)	1.25 (0.35, 4.52)	0.98 (0.15, 6.51)	0.97 (0.68, 1.37)
College or above	0.95 (0.62, 1.47)	0.77 (0.19, 3.08)	0.76 (0.13, 4.65)	1.01 (0.70, 1.45)
Maternal educational level (reference: junior high school or below)				
Senior high school	1.15 (0.80, 1.65)	1.07 (0.34, 3.33)	1.57 (0.33, 7.39)	1.13 (0.83, 1.53)
Junior college	0.91 (0.61, 1.36)	1.52 (0.43, 5.37)	5.95 (0.98, 36.12)	1.00 (0.72, 1.40)
College or above	0.99 (0.64, 1.52)	2.08 (0.50, 8.61)	4.34 (0.66, 28.47)	1.07 (0.74, 1.54)
Paternal occupation (reference: commerce and services)				
Professional and technical	1.01 (0.74, 1.36)	2.06 (0.77, 5.55)	0.54 (0.14, 2.14)	1.04 (0.81, 1.34)
Administrator and clerk	1.07 (0.79, 1.47)	**3.40 (1.20, 9.61)** ^*^	0.57 (0.17, 1.91)	1.02 (0.79, 1.31)
Other	1.02 (0.77, 1.36)	2.07 (0.88, 4.84)	2.01 (0.49, 8.23)	1.18 (0.93, 1.50)
Maternal occupation (reference: commerce and services)				
Professional and technical	0.75 (0.55, 1.02)	0.60 (0.23, 1.56)	2.87 (0.61, 13.62)	**0.75 (0.59, 0.97)** ^*^
Administrator and clerk	0.85 (0.59, 1.22)	0.45 (0.14, 1.49)	0.77 (0.20, 2.91)	0.79 (0.59, 1.07)
Housewife	0.93 (0.68, 1.27)	1.48 (0.52, 4.21)	**8.92 (1.28, 61.99)** ^*^	0.95 (0.73, 1.24)
Other	0.83 (0.60, 1.14)	0.87 (0.33, 2.29)	0.68 (0.21, 2.28)	0.82 (0.63, 1.06)
Monthly household income (reference: < 8000 RMB)				
8000 to 15,000 RMB	0.88 (0.66, 1.17)	0.52 (0.22, 1.21)	1.72 (0.47, 6.38)	0.86 (0.68, 1.10)
≥ 15,000 RMB	0.87 (0.64, 1.18)	3.45 (0.96, 12.42)	0.71 (0.19, 2.72)	0.86 (0.66, 1.11)
Refused to answer	0.85 (0.66, 1.10)	1.15 (0.50, 2.65)	1.22 (0.41, 3.64)	0.83 (0.67, 1.02)
Paternal weight status (reference: normal weight)				
Overweight	1.19 (0.98, 1.45)	1.03 (0.55, 1.95)	1.47 (0.58, 3.73)	**1.22 (1.03, 1.44)** ^*^
Obese	1.10 (0.77, 1.57)	1.58 (0.60, 4.20)	2.01 (0.60, 6.74)	1.34 (1.00, 1.80)
Maternal weight status (reference: normal weight)				
Overweight	1.04 (0.77, 1.40)	1.01 (0.45, 2.31)	0.95 (0.36, 2.56)	1.05 (0.82, 1.34)
Obese	0.81 (0.40, 1.64)	3.67 (0.38, 35.88)	0.93 (0.15, 5.89)	0.83 (0.48, 1.45)
Personal perception of weight status (reference: normal weight)				
Overweight	-	1.69 (0.92, 3.09)	**4.34 (1.45, 12.98)** ^**^	**4.92 (3.79, 6.38)** ^**^
Obese	- ^a^	2.46 (0.27, 22.09)	**5.55 (1.35, 22.92)** ^*^	**10.69 (4.61, 24.82)** ^**^

Bold text: OR was significant at 0.05 level

Values were represented as OR and 95 % CIs derived from binary logistic regression analyses

^a^ The number of children who perceive themselves as overweight or obese was too small to be included in the analysis

^b^ The normal-weight group did not include the underweight children

^*^ *P* < 0.05; ^**^ *P* < 0.01 ﻿

## Discussion

This study assessed the current prevalence of overweight and obesity among children in Southern China and examined the early life and behavioral factors that influenced such prevalence. The weight-loss practices and the factors associated with their adoption were also investigated.

A relatively high prevalence of overweight (11.2 %) and obesity (10.0 %), which were both higher than that in 2011 (10.5 and 7.5 %, respectively) [23], was observed among the study population. After standardizing based on the age-gender distribution of the population in Ma’s study [23], the standardized prevalence of obesity in our study (8.8 %) was still higher than that in 2011, indicating that the prevalence of childhood obesity has been increasing from 2007 to 2013. A further comparison showed that the prevalence of overweight and obesity in Guangzhou in 2013 were still lower than the average values of other large coastal cities in China (20.7 and 17.8 % in boys and 11.1 and 9.8 % in girls), but exceeded the national level (14.2 and 9.4 % in boys and 7.7 and 5.4 % in girls) in 2010 [4]. The disparity between Guangzhou and other large coastal cities may be attributed to the complex interaction of geographic-climate factors (warm and humid climate in Guangzhou) as well as different appetites (not greasy or strongly flavored) and dietary habits of children (drinking soup before eating) [3]. However, another study indicated that further economic development and lifestyle assimilation may reduce the “south–north gap” [30].

A higher prevalence of overweight or obesity was observed among boys than girls, which was consistent with previous findings in China [3, 13, 14, 31]. Socio-cultural, socio-economic, behavioral, and genetic factors may play important roles in gender disparity of obesity [31]. In Chinese culture, obesity in boys is recognized as strong, whereas Chinese girls prefer a slender shape and are more likely to control their weight compared with boys [31]. Behavioral factors may also contribute to the gender disparity. As presented in our study, boys consumed more fried food, drank more SSBs and were more likely to spent > 2 h per day of screen time (TV and computer) than girls (all *P* < 0.05), indicating that boys were more likely to engage in unhealthy behaviors. Children with and without siblings showed no difference in their weight status in the present study. A European study [32] revealed that singletons were more likely to be overweight than their peers with siblings when controlling for factors related to childhood overweight. However, the sample in the European study [32] included less than 30 % of singletons, which might contribute to the discrepancy between the current study and this one.

Recently, some studies have supported an influence of intrauterine development on risk of health and disease later in life [33]. Birth weight was generally considered as an indicator of gestational nutritional status [34]. In the present study, high birth weight (≥4.0 kg) was identified as a risk factor for obesity among children (adjusted OR: 2.34; 95 % CI: 1.53–3.58), which was consistent with previous findings [17, 35]. Nevertheless, Chinese pregnant women tend to be urged to consume a large amount of high-nutrition food (e.g., meat, eggs and nuts), which may result in fetal excessive weight gain. It was observed that the proportion of macrosomia increased from 3.2 % in 1985 to 4.3 % in 2005 in healthy term newborns in China [36]. In our study, the percentage of macrosomia was even higher (6.2 %). Considering higher risk of childhood obesity, mothers during pregnancy should keep a healthy diet to avoid infants with high birth weight. On the other hand, the role of low birth weight in the subsequent development of overweight and obesity was still unclear [17]. Compared with normal birth weight (2.5 ~ 4.0 kg), low birth weight (<2.5 kg) presented no significant association with overweight or obesity in our study. Furthermore, gestational age < 37 weeks was positively associated with overweight (versus 37 ~ 42 weeks, adjusted OR: 1.75; 95 % CI: 1.01–3.05) independent of birth weight, which might be explained by infant accelerated weight gain in premature. Previous findings regarding the relationship between gestational age and obesity remained inconsistent [37, 38]. Li et al. suggested that preterm large for gestational age (OR: 2.75) and preterm appropriate for gestational age (OR: 1.56) were both recognized as significant risk factors of childhood overweight and obesity [37]. The above study failed to demonstrate a relationship between preterm small for gestational age and later overweight or obesity [37]. More studies are need to analyze the independent associations between gestational age and obesity. We found no association between cesarean delivery and childhood obesity, on which the findings remained controversial in China [18, 39]. Although a protective effect of breastfeeding for subsequent obesity of children was demonstrated in several studies [19, 40], we found no statistically relationship between feeding patterns and overweight or obesity. However, the lowest prevalence of overweight and obesity was found among children with a breastfeeding duration of 4.0 ~ 5.9 months. A similar pattern was observed in a case-control study in Europe [41] and a cohort study in Brazil [42]. Consistent with other studies [13, 14], we found that children with a parent being overweight or obese were more likely to be overweight or obese compared with those with normal-weight parents. Parental overweight or obesity might have an influence on child’s obesity through genetic or behavioral factors such as dietary habits and family lifestyle.

We also assessed the associations of behavioral factors with overweight/obesity. Among the dietary behaviors, SSBs intake and eating speed faster than peers were positively associated with childhood overweight and obesity. The findings on SSBs intake were supported by previous studies [43, 44]. Given that SSBs intake was a significant contributor to weight gain [45], future obesity intervention strategies should focus on the consumption of SSBs among children. We proposed that schools should restrict the supply of soft drinks. Eating speed was another independent factor that affected overweight/obesity, which was consistent with the findings of other studies [15, 46]. Eating speed faster than peers (versus “as fast as peers”) showed a strong positive association with both overweight (adjusted OR: 1.76; 95 % CI: 1.27 – 2.43) and obesity (adjusted OR: 3.18; 95 % CI: 2.28–4.44), whereas eating speed slower than peers (versus “as fast as peers”) demonstrated an opposite association (adjusted OR: 0.55; 95 % CI: 0.36–0.84 and adjusted OR: 0.55; 95 % CI: 0.34–0.91, respectively). Previous studies found that the energy intake per day can significantly increase along with an increasing eating speed [47]. Among all dietary behaviors, eating speed demonstrated the strongest association with overweight and obesity, which indicated that this factor may play a crucial role in energy intake. Therefore, the eating speed of children should be moderated by launching health education programs. Short sleep duration was also considered a potential risk for overweight/obesity in children [48]. In our study, we found sleep duration as “9.0 ~ 11.0 h” (versus 7.0 ~ 8.9 h) was negatively associated with overweight (adjusted OR: 0.60; 95 % CI: 0.43–0.84), but not with obesity.

With regard to sedentary time, we investigated the relationships of single types of inactivity with obesity and found that daily homework time was independently associated with obesity, which was consistent with previous studies [11]. However, unlike previous findings [49, 50], television viewing was not associated with overweight or obesity. We hypothesized that children of this age can only begin to demonstrate excessive weight gain after a longer duration of television viewing. Sedentary behavior of Chinese children, including screen time and homework time, had increased over the last decade [51]. The unique Chinese culture, in which parents hold high expectations on the academic performance of their children, may influence and differentiate the sedentary behavior of Chinese children from those of children in developed countries [51]. Additional research are warranted to confirm the effect of doing homework on the prevalence of obesity among Chinese children.

It is well known that decreased physical activity was positively associated with increased BMI [52]. However, we did not observe significant associations between physical activities and overweight or obesity, which might be attributed to the lack of physical activities among the study participants. The majority of our participating children, either normal-weight or overweight, failed to meet the recommended 60 min of moderate- to vigorous-intensity physical activity daily [53]. A hypothesis is that these children might be exposed further to physical activity before we can observe any effect. Wang et al. [54] objectively assessed the physical activity levels of 2163 9–17-year-old Chinese children by using accelerometers in 2011 and found that only 9.4 % of boys and 1.9 % of girls reached the recommendation [53]. Intervention strategies must be proposed to promote the participation in physical activities.

Previous studies suggested that perceived overweight and the actual overweight status were strongly associated with weight-loss practices [55]. In our study, overweight/obese children were more likely to adopt weight-loss practice than normal-weight children, and the perception of weight status was strongly associated with the adoption of weight-loss practice among obese children as well as the entire population. Logistic regression analyses showed that the odds of adopting weight-loss practice were 4.9 and 10.7 times as high among children who perceived themselves as overweight and obese, respectively, which indicated that such perception was one of the major driving forces for children to lose weight. Nevertheless, a previous study [56] revealed that Chinese children tended to underestimate overweight and obesity status, particularly among children with high BMI. Therefore, an important first step of obesity intervention might be promoting accurate identification and interpretation of actual weight status in overweight and obese children.

This study presents several limitations. First, the collected data were cross-sectional, which can preclude inferences about causality. Second, a cluster sampling method was used to recruit the participants, which can result in selection bias. Third, BMI was used to measure weight status, which was incapable of distinguishing between total lean mass and fat mass. However, BMI is highly, positively correlated with fat mass in children [57]. So high BMI can be a fairly good indicator of excess body fatness in children. Fourth, the behavioral variables, such as dietary intake of certain foods, duration of physical activities and duration of sedentary behavior, were self-reported, so recall bias may exist. Besides, we did not use previously validated questionnaires to measure behavioral factors, which make the outcomes not straightforward to compare with those measured by the validated questionnaires.

## Conclusions

The prevalence of overweight (11.2 %) and obesity (10.0 %) among children in Guangzhou were higher than the previous reported level. High birth weight, SSBs intake, eating speed faster than peers, and homework time were positively associated with obesity among children. Therefore, future studies or programs should focus on the unhealthy dietary and sedentary behavior of children. An accurate perception of weight status plays an important role to improve the weight-loss practices in overweight and obese children.

## Abbreviations

BMI: Body mass index
CI: Confidence interval
OR: Odd ratio
SD: Standard deviation
SSBs: Sugar-sweetened beverages
WGOC: The Working Group of Obesity in China
